# Supplementary material for: Forecasting protein evolution by integrating birth-death population models with structurally constrained substitution models
Source: eLife. 2025 Sep 24;14:RP106365. doi: 10.7554/eLife.106365 (PMC12459951; doi:10.7554/eLife.106365)
Supplement: Supplementary file 1. [file elife-106365-supp1.pdf]

Supplementary File 1

Forecasting protein evolution by integrating birth-death population models with structurally constrained substitution models

The Supplementary File 1 includes Supplementary File 1A, Supplementary File 1B, Supplementary File 1C and references.

Supplementary Files 1-C

Supplementary File 1A. Evolutionary processes and corresponding parameters implemented in *ProteinEvolver2*. The user can specify a variety of parameters, optional or mandatory, to define an evolutionary scenario.

Evolutionary process	Parameter	Mandatory or optional | *Additional information*	
All	Number of replicates	M	
Evolutionary history	Birth-death process (includes parameters presented below)		*One of these options must be used*
Evolutionary history	Coalescent process (includes parameters presented below)	M	
Evolutionary history	Input phylogenetic tree/s (fixed tree in *Newick* format)		
DNA evolution / Substitution model of DNA evolution	Nucleotide frequencies	O	*By default, the JC model is applied*
DNA evolution / Substitution model of DNA evolution	Transition / transversion ratio	O	
DNA evolution / Substitution model of DNA evolution	Relative symmetrical substitution rates	O	
DNA evolution / Substitution model of DNA evolution	Relative asymmetrical substitution rates	O	
DNA evolution / Substitution model of DNA evolution	SCS models[1] for DNA	O	
Protein evolution / Substitution models of protein evolution	Empirical amino acid substitution model (it implements a variety of empirical models)[1]		*One of these options must be used*
Protein evolution / Substitution models of protein evolution	SCS models[2] for proteins	M	
Molecular evolution / Substitution models of evolution	Rate variation among sites[3]	O	
Molecular evolution / Substitution models of evolution	Proportion of invariable sites	O	
Molecular evolution / Substitution rate	Variable site-specific substitution rate	O	
Molecular evolution	User-specified sequence for the root node	O[4]	
Information in output files	Print sequences to a file	O	
Information in output files	Format of simulated multiple sequence alignments (*Fasta, Phylip, Nexus*)	O	*By default, printed in Phylip format*
Information in output files	Print sequence of the root node (GMRCA or MRCAs)	O	
Information in output files	Print simulated trees	O	
Information in output files	Print times of nodes of genealogies	O	
Information in output files	Print simulated ARG	O	
Information in output files	Print recombination breakpoints	O	
All	Simulation seed	O	
Information in the screen	Level of information printed on the screen	O	
Evolutionary history / Birth-death evolutionary process	Type of birth and death rates (specified or calculated)	M	*Birth and death rates*

			are specified or calculated from the fitness of the variant
Global birth-death rate variation among lineages following (Neher *et al*, 2014)	Model option for scenarios based on fitness, where death rate is 1 and birth rate is 1 + fitness	O	*Requires indicating No (1) or Yes (1)*
Evolutionary history / Birth-death evolutionary process	Type of ending the simulation of the birth-death process	M	*Reaching a specified sample size, number of tip nodes or evolutionary time*
Evolutionary history / Birth-death evolutionary process	Prune extinct nodes	O	
Evolutionary history / Birth-death evolutionary process	Outgroup and its branch length	O	
Evolutionary history / Birth-death evolutionary process	Substitution rate	M	
Evolutionary history / Birth-death evolutionary process	Effective population size; Haploid/Diploid	M	
Evolutionary history / Birth-death evolutionary process	Alignment length (nucleotides or amino acids)	M	
Evolutionary history / Coalescent evolutionary process	Sample size and alignment length (nt or aa)	M	
Evolutionary history / Coalescent evolutionary process	Effective population size; Haploid/Diploid	M	
Evolutionary history / Coalescent evolutionary process	Tip dates[5]	O	
Evolutionary history / Coalescent evolutionary process	Generation Time	O	
Evolutionary history / Coalescent evolutionary process	Exponential growth rate	O	
Evolutionary history / Coalescent evolutionary process	Demographic periods	O	
Evolutionary history / Coalescent evolutionary process	Migration model (island, stepping-stone, island-continent) and population structure	O	
Evolutionary history / Coalescent evolutionary process	Migration rate (constant or variable with time)	O	
Evolutionary history / Coalescent evolutionary process	Convergence of demes	O	
Evolutionary history / Coalescent evolutionary process	Homogeneous recombination rate	O	
Evolutionary history / Coalescent evolutionary process	Fixed number of recombination events	O	
Evolutionary history / Coalescent evolutionary process	Recombination hotspots	O	
Evolutionary history / Coalescent evolutionary process	Substitution rate	M	
Evolutionary history / Coalescent evolutionary process	Outgroup and its branch length	O	

Evolutionary history / User-specified phylogenetic tree/s	Number of input phylogenetic trees, alignment length (in nucleotides or amino acids) and rooted phylogenetic tree	M, M, M
Molecular evolution	Sequence length	M
Molecular evolution	Factor that multiplies the original substitution rate	O
Molecular evolution	Sequenced assigned to the root node	O
Molecular evolution / Structurally constrained substitution models of protein evolution	PDB file	M
Molecular evolution / Structurally constrained substitution models of protein evolution	Chain of the PDB file	M
Molecular evolution / Structurally constrained substitution models of protein evolution	Input file of amino acid contacts	M
Molecular evolution / Structurally constrained substitution models of protein evolution	Thermodynamic temperature	M
Molecular evolution / Structurally constrained substitution models of protein evolution	Configurational entropy per residue (unfolded)	M
Substitution models of protein evolution	Configurational entropy per residue (misfolded)	M
Molecular evolution / Structurally constrained substitution models of protein evolution	Configurational entropy offset (misfolded)	M
Molecular evolution / Structurally constrained substitution models of protein evolution	Third cumulant in REM calculation	M
Molecular evolution / Structurally constrained substitution models of protein evolution	Type of SCS model (Neutral or Fitness)	M
Molecular evolution / Structurally constrained substitution models of protein evolution	Effective population size for the fitness SCS model	O
Molecular evolution / Structurally constrained substitution models of protein evolution	Consideration of branch lengths	O
Molecular evolution / Structurally constrained substitution models of protein evolution	Amount of information about SCS models printed as output files	O

[1]A variety of empirical substitution models of protein evolution are implemented: *Blosum62* (Eddy, 2004; Henikoff & Henikoff, 1992), *CpRev* (Adachi *et al*, 2000), *Dayhoff* (Dayhoff *et al*, 1978), *DayhoffDCMUT* (Kosiol & Goldman, 2005), *FLU* (Dang *et al*, 2010), *HIVb* (Nickle *et al*, 2007), *HIVw* (Nickle *et al.*, 2007), *JTT* (Jones *et al*, 1992), *JonesDCMUT* (Kosiol & Goldman, 2005), *LG* (Le & Gascuel, 2008), *Mtart* (Abascal *et al*, 2007), *Mtmam* (Yang *et al*, 1998), *Mtrev24* (Adachi & Hasegawa, 1996), *RtRev* (Dimmic *et al*, 2002), *VT* (Muller & Vingron, 2000), *WAG* (Whelan & Goldman, 2001) or any user-specified matrix for all the sites or for every site (thus differing among sites).

[2]SCS models can be neutral or fitness-based landscape.

[3]Shape of the gamma distribution.

[4]If not specified, a random sequence is assigned to the root node according to the used nucleotide, codon or amino acid frequencies.

[5]In presence of convergence of demes, the tip nodes must be older than the convergence of demes.

Supplementary File 1B. Longitudinal data of the HIV-1 PR used to evaluate the accuracy of the forecasting protein evolution. For each patient, the first column indicates the identifier code (ID) of the patient in the Specialized Assistance Services in Sexually Transmissible Diseases and HIV/AIDS in Brazil. The next columns indicate, for every consensus sequence collected at a time *T* from the patient, the GenBank accession code and the number of amino acid substitutions accumulated since *T1* (shown in parenthesis). The last column indicates the HIV-1 PR inhibitor/s that the patient received.

**Patient ID**	**Longitudinal sample (*T*)**					**HIV-1 PR inhibitors administrated**
	T1 origin (# of substitutions)	***T2 (# of substitutions)***	***T3 (# of substitutions)***	***T4 (# of substitutions)***	***T5 (# of substitutions)***	
99842856	ON983124 (0)	ON983123 (3)	ON983126 (9)	ON983124 (11)	-	RTV, LPV
99943945	ON982892 (0)	ON982893 (5)	ON982894 (8)	ON982891 (11)	-	RTV, FPV
10701966	ON982841 (0)	ON982837 (6)	ON982838 (12)	ON982839 (17)	ON982840 (19)	ATV, RTV, LPV
12887	ON982884 (0)	ON982883 (3)	ON982885 (6)	ON982886 (8)	-	DRV, RTV
99817844	ON982842 (0)	ON982843 (5)	ON982845 (9)	ON982844 (12)	-	ATV, RTV
99654931	ON983078 (0)	ON983079 (10)	ON983077 (19)	ON983110 (22)	-	LPV
99574196	ON982995 (0)	ON983050 (10)	ON982996 (17)	ON982994 (19)	-	LPV
37000881	ON983034 (0)	ON983035 (1)	ON983036 (6)	ON983033 (8)	-	ATV, RTV, LPV
99412571	ON983119 (0)	ON983120 (3)	ON983121 (5)	ON983122 (7)	-	-
99783386	ON982927 (0)	ON982925 (4)	ON982924 (9)	ON982928 (12)	ON982926 (17)	LPV, RTV, ATV
23400093	ON983128 (0)	ON983130 (12)	ON983129 (18)	ON983127 (22)	-	DRV, RTV, ATV, LPV
4300388	ON982876 (0)	ON982875 (5)	ON982877 (11)	ON982878 (20)	-	LPV, DRV, RTV

Supplementary File 1C. Structures of the HIV-1 MA, SARS-CoV-2 Mpro, Influenza NS1 protein and, SARS-CoV-2 PLpro. Also structures of the HIV-1 PR selected as templates in homology modeling. The data of the HIV-1 matrix (MA) protein, influenza NS1 protein, and SARS-CoV-2 main protease (Mpro) and papain-like protease (PLpro) involved only one consensus sequence at the initial time, thus only one protein structure was used and did not require homology modelling because the study sequence was already present in an available protein structure. However, the HIV-1 protease (PR) dataset involved several independent HIV-1 populations (patients) and, a structural template was selected for each one. For each protein, the table shows the corresponding PDB code and protein chain used for the study. For the HIV-1 PR, the table presents the modelling quality and sequence identity of the structural templates for homology modelling (their coverage was 100%).

**HIV-1 MA**	
PDB code	*Protein chain*
7JXR	B
**SARS-CoV-2 Mpro**	
PDB code	*Protein chain*
7N8C	A
**SARS-CoV-2 PLpro**	
PDB code	*Protein chain*
6XA9	A
Influenza NS1	
PDB code	*Protein chain*
4OPH	A

**HIV-1 PR**				
Patient ID	*PDB code*	*Protein chain*	*Modelling quality*	*Sequence identity*
99842856	3LZS	A	555.3381	88.889
99943945	1C6X	A	453.8610	83.838
10701966	3U71	A	467.8960	95.960
12887	3EKP	A	447.4938	80.808
99817844	1HIV	A	641.8560	90.816
99654931	3LZS	A	464.9483	90.909
99574196	1AID	A	632.7499	93.939
37000881	1AID	A	544.9096	92.929

99412571	3D3T	A	529.7580	90.909
99783386	2FDE	A	450.4926	94.949
23400093	1SGU	A	435.4226	91.818
4300388	2FDD	E	638.4982	83.838

References cited in the supplementary file

Abascal F, Posada D, Zardoya R (2007) MtArt: A New Model of Amino Acid Replacement for Arthropoda. *Mol Biol Evol* 24: 1-5

Adachi J, Hasegawa M (1996) MOLPHY version 2.3: programs for molecular phylogenetics based in maximum likelihood. *Comput Sci Monogr* 28: 1-150

Adachi J, Waddell PJ, Martin W, Hasegawa M (2000) Plastid genome phylogeny and a model of amino acid substitution for proteins encoded by chloroplast DNA. *J Mol Evol* 50: 348-358

Dang CC, Le QS, Gascuel O, Le VS (2010) FLU, an amino acid substitution model for influenza proteins. *BMC Evol Biol* 10: 99

Dayhoff MO, Schwartz RM, Orcutt BC (1978) A model of evolutionary change in proteins. In: *Atlas of protein sequence and structure*, Dayhoff M.O. (ed.) pp. 345-352. Washington D. C.

Dimmic MW, Rest JS, Mindell DP, Goldstein RA (2002) rtREV: an amino acid substitution matrix for inference of retrovirus and reverse transcriptase phylogeny. *J Mol Evol* 55: 65-73

Eddy SR (2004) Where did the BLOSUM62 alignment score matrix come from? *Nat Biotechnol* 22: 1035-1036

Henikoff S, Henikoff JG (1992) Amino acid substitution matrices from protein blocks. *Proc Natl Acad Sci U S A* 89: 10915-10919

Jones DT, Taylor WR, Thornton JM (1992) The rapid generation of mutation data matrices from protein sequences. *Comput Appl Biosci* 8: 275-282

Kosiol C, Goldman N (2005) Different versions of the Dayhoff rate matrix. *Mol Biol Evol* 22: 193-199

Le SQ, Gascuel O (2008) An improved general amino acid replacement matrix. *Mol Biol Evol* 25: 1307-1320

Muller T, Vingron M (2000) Modeling amino acid replacement. *Journal of Computational Biology* 7: 761-776

Neher RA, Russell CA, Shraiman BI (2014) Predicting evolution from the shape of genealogical trees. *Elife* 3

Nickle DC, Heath L, Jensen MA, Gilbert PB, Mullins JI, Kosakovsky Pond SL (2007) HIV-specific probabilistic models of protein evolution. *PLoS One* 2: e503

Whelan S, Goldman N (2001) A general empirical model of protein evolution derived from multiple protein families using a maximum-likelihood approach. *Mol Biol Evol* 18: 691-699

Yang Z, Nielsen R, Masami H (1998) Models of amino acid substitution and applications to mitochondrial protein evolution. *Mol Biol Evol* 15: 1600-1611
